# Genotype-matched mapping reveals consistent regional flavour signatures and rhizosphere microbial correlates in spring-flush Yunnan large-leaf tea

**DOI:** 10.1016/j.fochx.2026.103869

**Published:** 2026-04-16

**Authors:** Jiayin Tong, Yunhan Li, Yanmei Zhang, Panpan Zhang, Kaibo Wang, Qian Zou, Shiquan Shen

**Affiliations:** a Tea Research Institute, Yunnan Academy of Agricultural Sciences, Kunming, Yunnan 650205, China; b Yunnan Key Laboratory of Tea Science, Yunnan Academy of Agricultural Sciences, Kunming, Yunnan 650205, China; c Yunnan Tea Innovation Center of Industry Technology, Yunnan Academy of Agricultural Sciences, Kunming, Yunnan 650205, China; d Univ. Bordeaux, INRAE, Bordeaux INP, Bordeaux Sciences Agri, UMR 1366, OENO, ISVV, F-33882, Villenave d'Ornon, France

**Keywords:** Rhizosphere microbiome, Tea terroir, *Camellia sinensis*, Volatilomics, Non-volatile chemistry, Genotype-matched design

## Abstract

Microbial correlates of tea terroir are often confounded by cultivar. Using eight SNP-confirmed genotype-matched pairs large-leaf tea cultivar pairs across Menghai and Pu’er, we tested whether rhizosphere communities covary with flavour chemistry during the spring flush. Under genotype control, the volatile Signature Index (VSI) was consistently higher in Menghai in all pairs, with a β-caryophyllene detection/non-detect contrast (7/8 detected in Pu’er vs 0/8 in Menghai). Non-volatiles chemistry showed a two-tier response: EGCG increased in Menghai, whereas nitrogen-associated taste allocation varied by pair.

Bulk soil nitrogen and enzyme activities did not directly explain leaf patterns, while bacterial and fungal communities showed significant regional separation and cross-kingdom concordance. Within this strict genotype-matched framework, the results identify spring-flush regional flavour signatures and their microbial correlates as association-level patterns, providing candidate targets for subsequent mechanistic study.

## 1 Introduction

Premium teas derive much of their market value from their provenance-linked sensory distinctiveness. Even when manufacturing styles are broadly similar, teas produced in different geographic regions often show reproducible differences in aroma composition and overall volatile profiles, providing a strong basis for the use of volatilomics in authenticity and origin assessment (Yang et al., 2013; Ye et al., 2012). At the same time, tea aroma and taste are shaped not only by the growing environment but also by factors such as harvest timing and post-harvest processing, both of which can influence volatile and non-volatile components (Ho et al., 2015; Ao et al., 2024, Huang et al., 2023, Ouyang et al., 2025, Yang, Baldermann and Watanabe, 2013, Zhu et al., 2025). As a result, regional differences are often difficult to attribute with confidence, because environmental effects are frequently entangled with planting material, local agronomy, and small differences in processing.

Among pre-harvest factors, the rhizosphere microbiome has been proposed as a potential component of terroir because microbial communities respond to edaphic and climatic gradients and can vary with plant physiological status. In perennial crops such as grapevines, studies have reported regional differences in microbial biogeography and soil-plant microbiome coupling, giving rise to the broader concept of microbial terroir (Bokulich et al., 2016; Gilbert et al., 2014). Tea systems also show potential for microbial contributions to flavour, but they present methodological challenges. Field studies often lack consistent cultivar control and clear units of inference, and cultivar–environment confounding frequently limits the strength of regional comparisons. Consequently, although geographic microbiome separation is often reported, it remains unclear whether these patterns primarily reflect environmental context, planting material, or their interaction.

To address this problem, we used a genotype-matched field design within a controlled spring-flush sampling window. Eight widely cultivated large-leaf tea cultivars were selected from existing plantings in two major tea production hubs in Yunnan (Menghai and Pu’er). These cultivars were confirmed as genotype-matched pairs through genotyping. Each genotype-matched pair was treated as the independent unit of inference (*n* = 8 pairs), and all statistical analyses were designed to respect this pairing. This design reduces cultivar–environment confounding while maintaining realistic field conditions under standardized harvest and processing. More broadly, it may offer a useful framework for microbial terroir studies in perennial crops, where regional patterns are often complicated by mixed genotype composition and uneven planting histories.

To connect microbial community patterns with interpretable flavour traits, we defined two a priori composite indices with fixed variable sets and equal weighting. The non-volatile index (NPI) captures the balance between a phenolic/alkaloid pool (major catechins and caffeine) and a nitrogenous/amino taste pool (theanine and GABA). Because nitrogen allocation is tightly linked to shoot growth, this index may be sensitive to residual phenological differences even under standardized harvesting conditions. The volatile signature index (VSI) was pre-specified to summarize the balance between two fixed volatile sets. Specifically, VSI increases when the floral/fruity set shows higher standardized abundance and/or the resinous/woody set shows lower standardized abundance, so that larger VSI values indicate a shift toward a monoterpene-enriched profile and away from resinous/woody volatiles (Ho et al., 2015; Yang et al., 2013). Using pre-specified composite indices also reduces reliance on post hoc feature selection from high-dimensional flavour data and provides biologically interpretable summary axes for matched-pair comparisons.

As a functional layer linked to soil processes, we measured rhizosphere enzyme activities (urease, sucrase/invertase, acid phosphatase, catalase, and β-glucosidase), edaphic properties, and amplicon-based bacterial and fungal communities. Volatile compounds were profiled by HS-SPME–GC–MS using pre-defined detection and blank-filtering rules consistent with standard approaches in tea volatilomics and origin discrimination (Yang et al., 2013; Ye et al., 2012). Samples were processed in randomized order with interleaved blanks and pooled QC controls to monitor analytical stability.

Within this genotype-matched spring-flush snapshot, we test two association-focused hypotheses. First, rhizosphere community structure would show pair-consistent cross-terroir covariation with VSI/NPI and electronic-tongue taste readouts, with rhizosphere enzyme activities included as covariates to assess whether any measured functional phenotype aligned with flavour contrasts at the sampled resolution. Second, a subset of prevalent and abundant genera would show consistent cross-terroir directionality across cultivar pairs, thereby providing candidates for multi-season validation and mechanistic study. With eight independent genotype-matched pairs, the study was designed to assess not only whether regional contrasts are consistent within this spring-flush snapshot, but also whether the observed signal recurs across pairs rather than reflecting pair-specific variation.

In this study, microbial terroir refers to region-structured rhizosphere community fingerprints observed under standardized harvest and processing conditions within a spring-flush window. Mapping and linking are used to describe paired association and recoverability analyses within genotype-matched pairs rather than causal inference. Volatile anchors are treated as robust candidate markers within the boundary conditions of the present study.

## 2 Materials and methods

### 2.1 Study site and cultivar selection

The study was conducted in two major production hubs for large-leaf teas in Yunnan, China: Menghai (approx. 23°24′ N, 100°27′ E; elevation ∼1200 m; subtropical monsoon climate; annual precipitation ∼1500 mm) and Pu’er (approx. 22°47′ N, 101°00′ E; elevation ∼1300 m; broadly comparable climate but distinct edaphic context, including higher clay content). These two sites provide an edaphic contrast under shared germplasm and harmonized field management, enabling genotype-controlled assessment of microbial–terroir covariation.

Eight widely planted cultivars (Yunkang-10, Xueya-100, Pujing, Yungui, Zijuan, Aifeng, Duanjiebaihao, and QianMei-601) were established as parallel plantings at both sites in the same planting year. At the time of sampling, all tea plants were 13 years old, and the parallel plantings had therefore been maintained for 13 years. To support the integrity of the genotype-matched design, cultivar identity and cross-site pairing were confirmed using the TEA5K liquid-phase multiple-SNP (mSNP) array based on the Genotyping by Target Sequencing (GBTS) system, which has been previously validated in modern tea cultivars (Liu et al., 2025). Based on prior platform validation, a GS threshold of 90% was used for same-cultivar assignment, and this criterion was applied in the present study to confirm cultivar identity and cross-site pairing.

For each cultivar × site, 10–15 randomly selected trees were sampled and pooled as a composite to reduce within-site micro-heterogeneity. For cultivar i (*i* = 1–8), a matched pair was defined as (M_i_, P_i_), where M_i_ denotes the Menghai composite and P_i_ the corresponding Pu’er composite of the same cultivar. All inferential statistics were conducted at the pair level (*n* = 8 pairs); figures may display all 16 composites for visualization.

### 2.2 Sampling and standardized green-tea processing

Fresh shoots were harvested at one bud with two leaves stage on April 24, 2025, with sampling completed on the same day at both sites. The harvested material was processed into a standardized green-tea product within 24 h of harvest. All cultivar × region composites followed the same pilot-scale protocol to minimize post-harvest variability and to enable comparisons that primarily reflect pre-harvest growing conditions. In parallel, an aliquot of fresh leaves was flash-frozen in liquid nitrogen and stored at −80 °C for complementary analyses; frozen material was not used for tea manufacturing.

Leaves were withered in a climate-controlled chamber at 25 °C and 70% relative humidity for 6 h. Enzyme inactivation (“kill-green”) was performed by pan-firing at 230 °C for 2 min using an electric wok (DL-6CST-100, Fujian, China), followed by rolling for 30 min using a tea rolling machine (DL-6CRT-50, Fujian, China). Leaves were then dried at 80 °C for 4 h to a final moisture content <5% (*w*/w) using a temperature-controlled drying oven (GZX-9240MBE, Boxun, Shanghai, China).

Finished teas were milled (FW135 grinder, Taisite, Tianjin, China), passed through a 40-mesh sieve, sealed in airtight containers, and stored at −20 °C until chemical analyses. Processing order across cultivar pairs was randomized, using identical settings and predefined endpoints.

### 2.3 Rhizosphere soil collection

Fine roots (0–20 cm depth) were excavated around target trees. After gently shaking off loosely bound bulk soil, rhizosphere soil adhering to fine roots (approx. 1–3 mm) was brushed into sterile tubes. Field blanks were collected onsite; tools were sterilized with 70% ethanol between trees; gloves were changed between plots; and samples were transferred to portable cold storage within 4 h of collection.

For each cultivar at each site, three biological replicate plots were randomly selected from the corresponding planting area. Within each replicate plot, three rhizosphere soil subsamples were collected and pooled to form one plot-level composite sample. The three plot-level composite samples were then thoroughly mixed to generate one final matched composite rhizosphere soil sample for each cultivar × site. Upon return to the laboratory, each final composite sample was split immediately: aliquots for DNA extraction were stored at −80 °C; aliquots for enzyme assays were refrigerated and processed within a specified time window; and aliquots for physicochemical analyses were air-dried and sieved. Extraction blanks and sequencing controls were processed alongside samples.

### 2.4 Soil physicochemical properties

Air-dried soils were sieved to 2 mm (or 0.149 mm for total nutrient and organic matter assays). Soil physicochemical properties were measured following national standard methods: pH (1:2.5 soil:water; NY/T 1121.2–2006), total nitrogen (TN, Kjeldahl; NY/T 1121.24–2012), total phosphorus (TP, molybdenum–antimony colorimetry; NY/T 88–1988), available N (alkali diffusion; LY/T 1228–2015), available P (Olsen; NY/T 1121.7–2014), available K (ammonium acetate; NY/T 889–2004), and organic matter (dichromate oxidation; NY/T 1121.6–2006).

Method blanks, duplicate samples, and certified reference materials (where applicable) were included for quality control. Batches failing QC checks were re-assayed, and only QC-qualified measurements were used for downstream analyses.

### 2.5 Soil enzyme activities

Five rhizosphere enzyme activities—urease (S-UE, μg/d/g), sucrase (S-SC, mg/d/g), acid phosphatase (S-ACP, nmol/h/g), β-glucosidase (S-β-GC, nmol/h/g), and catalase (S-CAT, μmol/h/g)—were measured using commercial assay kits (Solarbio Science & Technology Co., Ltd., Beijing, China) according to the manufacturer's instructions. Enzyme activities were summarized at the cultivar × region composite level and considered in sensitivity analyses.

### 2.6 Non-volatile chemistry

#### 2.6.1 Catechins and caffeine (HPLC–DAD/UV)

Catechins (GA, GC, EGC, C, EC, EGCG, GCG, ECG, and CG) and caffeine were determined following GB/T 8313–2018. Dried tea samples were milled to a homogeneous powder and extracted with 70% methanol according to the standard protocol. Reference standards of catechins (GA, GC, EGC, C, EC, EGCG, GCG, ECG, and CG) were purchased from Shanghai Yuanye Biotechnology Co., Ltd., and the caffeine standard was purchased from Chengdu Desite Biotechnology Co., Ltd. Compound identification was based on comparison of sample peak retention times with those of the corresponding single-component reference standards. Quantification was carried out using calibration curves established from mixed standard solutions at different concentration levels. Analyses were performed on an LC-5090 HPLC system equipped with a diode-array detector (DAD) and an autosampler (Zhejiang Fuli Analytical Instrument Co., Ltd., China). Separations were carried out on a Sunniest C18 column (250 × 4.6 mm, 5 μm) maintained at 35 °C. The flow rate was set at 1.0 mL/min.

Mobile phase A was an aqueous solution containing 9% acetonitrile, 0.3% acetic acid, and 0.2% EDTA-2Na, and mobile phase B was an aqueous solution containing 80% acetonitrile, 0.3% acetic acid, and 0.2% EDTA-2Na. The gradient program was 0–10 min, 100% A; 10–35 min, linear to 68% A/32% B; 35–40 min, held at 68% A/32% B; 40–40.1 min, ramped to 20% A/80% B; 40.1–50 min, held at 20% A/80% B; and 50.1–65 min, re-equilibration at 100% A. Detection was monitored at 278 nm, with an injection volume of 10 μL.

#### 2.6.2 Free amino acids (ion-exchange chromatography with post-column derivatization)

Free amino acids, including theanine and GABA, were determined following GB/T 30987–2020 using an automated amino acid analyzer (Svkam S-433, manufactured in Germany). Samples were extracted with boiling water, filtered, and injected (50 μL) onto a lithium-type cation-exchange column (LCA K0.7/Li). The column temperature was programmed from 37 °C to 74 °C, followed by post-column ninhydrin derivatization at 130 °C. The elution and derivatization pump flow rates were 0.45 and 0.25 mL/min, respectively.

Detection was performed at 440 nm for proline and hydroxyproline and at 570 nm for other amino acids. Quantification used external calibration with a mixed standard containing theanine (500 nmol/mL) and 34 amino acids (each 100 nmol/mL; including GABA; Sichuan Weikeqi Biotechnology Co., Ltd). Results were calculated on a dry-matter basis (dry matter determined by GB/T 8303) and reported as mg/100 g.

### 2.7 Volatile profiling (HS-SPME–GC–MS)

Volatiles were profiled using headspace solid-phase microextraction coupled to GC–MS. Ground tea (0.20 g) was placed in a 20 mL headspace vial with 1.0 mL ultrapure water, 0.20 g NaCl, and 10 μL of ethyl decanoate internal standard (50 μg/mL in methanol). Samples were equilibrated at 60 °C for 20 min and extracted for 20 min using a 50/30 μm DVB/CAR/PDMS fiber. Blank vials and pooled QC samples were analyzed periodically throughout the analytical sequence.

Analyses were performed on an Agilent 7890B/5977B system equipped with an SH-5Sil-MS column (30 m × 0.25 mm × 0.25 μm) under EI mode (70 eV). The ion source and transfer line temperatures were 230 °C and 250 °C, respectively. The oven program was 40 °C (2 min), ramped at 2 °C/min to 60 °C, 1 °C/min to 80 °C, 5 °C/min to 120 °C, and 3 °C/min to 180 °C (2 min).

Compound identification and data processing. Volatile features were annotated primarily at MSI level 2 by matching mass spectra to the NIST 20 library (match factor > 800) and by comparing linear retention indices (LRIs; calculated using a C7–C40 n-alkane standard mix) against literature values. Peak integration followed a pre-defined workflow with blank-based feature filtering. Raw peak areas were normalized to the total non-IS signal and expressed as area percentages (Area%). Where technical replicates were acquired (suffix “-1/−2”), Area% values were averaged prior to downstream analyses. A compound was considered “detected” only if a chromatographic peak could be integrated and the corresponding feature passed the pre-defined blank-based filtering and annotation criteria (NIST match factor and LRI agreement); otherwise, it was treated as “not detected” under this pipeline and recorded as 0 (not imputed). Paired detect/non-detect contrasts were assessed using McNemar's exact test (§2.12).

### 2.8 Electronic tongue

Taste attributes (sourness, bitterness, umami, astringency, and aftertaste) were profiled using an electronic tongue system (Insent SA402B plus, Insent Inc., Atsugi, Japan). Tea infusions were prepared by brewing 5 g of tea in 250 mL water (95 °C) for 5 min and cooling to room temperature prior to measurement. Sensors were conditioned and measurements were drift-corrected according to the manufacturer's standard operating procedure (SOP).

Each cultivar × region composite was measured in triplicate, and replicate readouts were averaged after routine outlier removal. Valid readouts were obtained for 15 of 16 composites; analyses were conducted on complete cases (*n* = 15).

### 2.9 DNA extraction, amplicon sequencing, and taxonomy

DNA was extracted from 0.25 to 0.50 g rhizosphere soil using the DNeasy PowerSoil Pro Kit (Qiagen, Hilden, Germany), with extraction blanks and a mock community processed in parallel. The bacterial 16S rRNA gene (V3–V4 region) was amplified using primers 338F/806R, and the fungal ITS1 region was amplified using primers ITS1F/ITS2R. Amplicons were sequenced on an Illumina MiSeq platform (2 × 250 bp). Reads were processed in QIIME2 (v2023.2) using DADA2 for quality filtering, denoising, and chimera removal to generate amplicon sequence variants (ASVs).

Taxonomy was assigned in QIIME2 using the RDP Classifier with a confidence threshold of 0.7. Bacterial ASVs were classified against the SILVA database (release 138.2), and fungal ASVs were classified against the UNITE database (release 9.0).

For diversity analyses, ASV tables were rarefied to a fixed sequencing depth (bacteria: 44,867 reads per sample; fungi: 41,725 reads per sample). Bray–Curtis dissimilarities were used for community-level comparisons, with CLR/Aitchison-based analyses performed as a compositional sensitivity check. Differential abundance was tested on non-rarefied counts using DESeq2 (design ∼ region + cultivar) with BH-FDR correction. Functional predictions (PICRUSt2) and fungal guild annotations (FUNGuild), where reported, were filtered using NSTI diagnostics and restricted to “probable/highly probable” confidence assignments, respectively. Soil enzyme activities (§2.5) were used as the primary functional measurements, with in silico annotations used as complementary summaries.

### 2.10 Composite indices (VSI and NPI)

To summarize flavour chemistry in a pathway-informed manner while avoiding post hoc axis construction, we defined two a priori composite indices as primary flavour axes before any outcome-aware analyses. All component variables were z-scored across the 16 cultivar × terroir composites prior to index calculation. Both indices use fixed variable sets and equal weighting, with no data-driven variable selection or weight tuning based on the present dataset. Robustness audits (alternative scaling, expanded/reduced variable sets, and alternative weighting schemes) were conducted as sensitivity analyses and were not used for primary inference.

These compounds were selected a priori to represent two interpretable flavour dimensions rather than to maximize separation in the present dataset. The variables were first organized into volatile and non-volatile layers, and then grouped within each layer into positive and negative sets according to their dominant sensory or flavour-chemical roles, with fixed equal weighting applied within each set.

$$\mathrm{VSI}=\frac{1}{n_{+}}\;\sum_{v\in V_{+}}z\left(v\right)-\frac{1}{n_{-}}\;\sum_{v\in V_{-}}z\left(v\right)$$

where V_+_ denotes the fixed floral/fruity volatile set, V_−_ denotes the fixed resinous/woody volatile set, n_+_ and n_−_ are the numbers of variables in V_+_ and V-, respectively, and z(v) is the z-scored value of variable v across the 16 cultivar × terroir composites.

$$\mathrm{NPI}=\frac{1}{m_{+}}\;\sum_{x\in X_{+}}z\left(x\right)-\frac{1}{m_{-}}\;\sum_{x\in X_{-}}z\left(x\right)$$

where X_+_ denotes the fixed phenolic/alkaloid pool, X- denotes the fixed amino/nitrogenous taste pool, m_+_ and m_−_ are the numbers of variables in X_+_ and X_−_, respectively, and z(x) is the z-scored value of variable x across the 16 cultivar × terroir composites.

Variable sets (fixed, equal-weight):

V_+_ (floral/fruity): linalool; geraniol; linalool oxide; decanal.

V_−_ (resinous/woody): β-Caryophyllene; 1-Octen-3-ol.

X_+_ (phenolic/alkaloid pool): caffeine; EGCG; ECG; EGC; EC.

X_−_ (amino / nitrogenous taste pool): theanine; GABA.

Under these definitions, higher VSI indicates a shift toward the floral/fruity volatile set and away from the resinous/woody set, whereas higher NPI indicates a higher phenolic/alkaloid contribution relative to the amino/nitrogenous taste pool.

All component variables were z-scored across the 16 cultivar × terroir composites prior to index calculation.

### 2.11 Core taxa definitions and threshold auditing

Genus-level relative abundance tables were constructed after standard QC and taxonomy assignment (§2.9). Prevalence was defined as the proportion of the 16 cultivar × terroir composites in which a genus had non-zero relative abundance (after applying identical filtering rules across all samples), and typical abundance was summarized as the median relative abundance across the 16 composites.

Primary core definition (v1.0). Core taxa were defined a priori as genera with (1) prevalence ≥70% and (2) median relative abundance ≥0.1% across the 16 composites. Genera with prevalence 30–70% were classified as intermittent, and those with prevalence <30% as transient.

Threshold audit (Robustness grid). To assess robustness to cutoff choice, prevalence thresholds were varied from 60% to 80% (5% increments) and abundance thresholds from 0.05% to 0.20% (0.05% increments). For each grid setting, shortlist stability was summarized by: (1) direction consistency, defined as the fraction of genotype-matched pairs showing the same sign of the within-pair difference (Menghai − Pu’er); and (2) rank concordance, defined as the Spearman correlation of genus effect-size ranks relative to the v1.0 definition, where effect size was defined as the within-pair difference in genus relative abundance.

Pair-restricted null. To contextualize stability under a small number of pairs (*n* = 8), terroir labels were permuted strictly within each cultivar-matched pair, and the audit procedure was repeated to generate null distributions for the stability metrics. Audit outputs are provided in Supplementary Files; the main text reports v1.0 results, using the audit strictly for robustness assessment.

### 2.12 Statistical analysis

All analyses respected the genotype-matched paired design (n = 8 pairs), treating the cultivar pair as the independent unit of inference.

Community-level analyses. Microbial β-diversity was quantified using Bray–Curtis dissimilarity. Unconstrained ordination used PCoA and constrained ordination used distance-based RDA (db-RDA). To respect the genotype-matched paired design, statistical significance for PERMANOVA (adonis2), betadisper, Procrustes tests, and db-RDA global models was assessed using a pair-restricted permutation scheme in which terroir labels were swapped only within each cultivar pair. Because the matched design yields a finite permutation space (2^8^ = 256 within-pair labelings), we report exact, design-consistent *P*-values (p_pair_) obtained by enumerating all 256 pair-restricted permutations.

Univariate analyses. Continuous traits (non-volatile chemistry, enzyme activities, e-tongue attributes, and composite indices) were tested on within-pair contrasts using paired *t*-tests for approximately symmetric differences and Wilcoxon signed-rank tests otherwise. For paired continuous variables, effect size was summarized using Cohen's dz., defined as the mean within-pair difference divided by the standard deviation of the within-pair differences, so that larger absolute dz. values indicate stronger paired contrasts. For volatile features evaluated as binary detect/non-detect outcomes (§2.7), paired contrasts were tested using McNemar's exact test. Multiple testing was controlled within endpoint families using the Benjamini–Hochberg FDR procedure.

Robustness and validation. CLR/Aitchison-based sensitivity analyses were performed for compositional checks where applicable. Where predictive modeling was performed, leave-one-pair-out (LOPO) cross-validation was used to prevent genotype leakage. Crucially, for composite indices (VSI/NPI) used within LOPO loops, standardization parameters were re-estimated within each training fold and applied to the held-out pair to maintain strict out-of-fold independence.

## 3 Results and discussion

### 3.1 Pair-consistent volatile shift and a β-caryophyllene detect/non-detect contrast

**Fig. 1:**
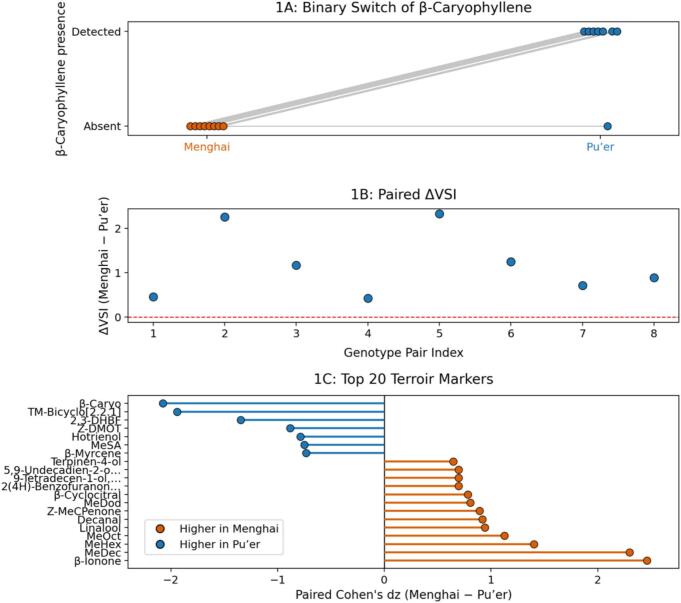
Volatile contrasts under genotype-matched pairing (*n* = 8 pairs).

Under strict genotype-matched pairing (*n* = 8 pairs), the volatile layer showed a uniform regional direction at the index level. The Volatile Signature Index (VSI) was higher in Menghai than in Pu’er for all matched comparisons (8/8). VSI contrasts a floral/fruity volatile set (linalool, geraniol, linalool oxide, and decanal) against a resinous/woody set (β-caryophyllene and 1-octen-3-ol). Across pairs, the paired difference ΔVSI (Menghai - Pu’er) had a median of 1.028 (IQR 0.646–1.50; Fig. 1B). This directionality was supported by an exact paired sign test (two-sided *P* = 0.0078) and a paired *t*-test (two-sided *P* = 0.0028), corresponding to a standardized paired effect size of d_z_ = 1.59 (bootstrap 95% CI [1.25, 3.02]; Table S1).

To interpret the index-level direction chemically, we used the signed decomposition of VSI (Methods; Table S1). In this definition, the positive VSI component is characterized by linalool-related terpenoids and allied oxygenated volatiles, whereas the negative component includes β-caryophyllene and 1-octen-3-ol. Consistent with the index-level direction, linalool (Area%) increased in Menghai in all 8/8 pairs (exact sign test P = 0.0078; Table S1), providing a continuous-direction compound anchor under the matched design.

By contrast, β-caryophyllene followed a detect/non-detect pattern under the pre-specified detection and blank-filtering workflow (Fig. 1A). It was detected in Pu’er in 7/8 matched pairs but was not detected under the applied rule in any matched Menghai sample (0/8; Table S1; Table S2). This paired discordance was supported by McNemar's exact test (two-sided *P* = 0.0156). Where detected in Pu’er, β-caryophyllene accounted for a small fraction of total signal (Area% ≈ 0.05–0.13; Table S1; Table S2), so under the current criteria the contrast is best treated operationally as a binary feature.

To contextualize the volatile feature space after preprocessing, Fig. 1C lists the top 20 features ranked by |d_z_| as a descriptive snapshot under the current pipeline rather than a confirmatory marker set (Fig. 1C; Methods; Table S1; Table S2).

A pathway-level interpretation is plausible. Linalool belongs to the monoterpene class commonly linked to plastidial isoprenoid metabolism, whereas sesquiterpenes such as β-caryophyllene are typically associated with cytosolic isoprenoid metabolism (Dudareva et al., 2013; Ho et al., 2015). The linalool increase observed in every genotype-matched contrast makes cultivar composition alone a less sufficient explanation for the observed directionality and is compatible with provenance-associated differences expressed at the volatile-pathway level under pairing. This reading is also consistent with the broader tea-aroma literature, which identifies terpene and oxygenated volatile blocks as important contributors to regional aroma differences while remaining responsive to physiological state and processing context (Ho et al., 2015; Kato & Shibamoto, 2001; Lee et al., 2013; Yang et al., 2013).

At the same time, the β-caryophyllene pattern warrants a measurement-aware reading. Sensomics and GC-O studies emphasize that GC–MS Area% is not a direct surrogate for sensory contribution because odor thresholds and matrix effects can dominate odor activity (Kumazawa & Masuda, 2002; Wang et al., 2025; Wei et al., 2024). In this light, β-caryophyllene is best treated as an operational provenance marker under the current detection/blank-filtering rule rather than as definitive evidence of biochemical on/off regulation. Within the present association-level framework, one plausible interpretation is that region-structured rhizosphere conditions may contribute to shifts in precursor availability or physiological context, thereby influencing the relative balance between monoterpene- and sesquiterpene-related volatile outputs, although the present dataset does not resolve the underlying mechanism. Having established that the volatile layer provides the most directionally invariant signal under genotype matching, we next examine whether non-volatile chemistry exhibits comparable pair-consistent directionality under the same design.

(A) β-caryophyllene shows a detect/non-detect asymmetry (Pu’er 7/8 vs Menghai 0/8; McNemar's exact *P* = 0.0156). Grey lines connect matched pairs.

(B) Paired ΔVSI (Menghai - Pu’er) is positive in all pairs (8/8; sign test *P* = 0.0078; paired *t*-test *P* = 0.0028); dashed line, ΔVSI = 0.

(C) Volatile features ranked by signed paired effect size (Cohen's d_z_; Menghai - Pu’er); the top 20 is a descriptive ranking under the current preprocessing.

### 3.2 Stable catechin/caffeine directionality with variable theanine allocation

**Fig. 2:**
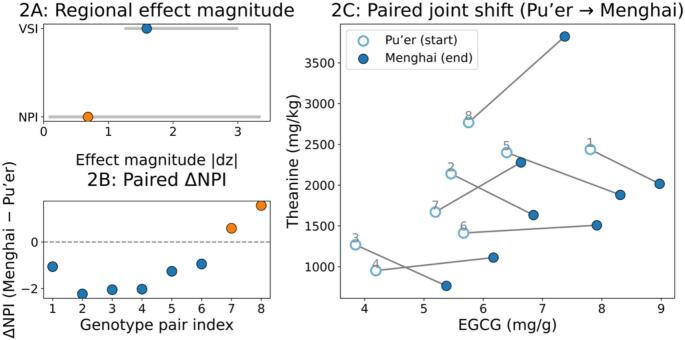
Non-volatile responses under genotype-matched pairing (*n* = 8 pairs).

At the composite level, the non-volatile axis is not directionally invariant under pairing. NPI contrasts a phenolic/alkaloid pool (caffeine, EGCG, ECG, EGC, and EC) against an amino/nitrogenous taste pool (theanine and GABA). Across the eight pairs, ΔNPI (Menghai − Pu’er) was negative in 6/8 pairs and positive in 2/8 pairs (median ΔNPI = −1.16; Fig. 2B), and the paired Wilcoxon signed-rank test did not support a cohort-level shift (two-sided *P* = 0.1094). Consistent with this sign instability, the magnitude of the NPI effect (|d_z|_) was generally smaller than that observed for VSI when the two indices are compared on the same paired-effect scale (Fig. 2A).

Disaggregating NPI reveals that this aggregate instability arises from heterogeneous behavior of its constituents. Several components were strictly directional under the same pairing scheme: EGCG and caffeine were higher in Menghai in 8/8 pairs (exact sign test *P* = 0.0078; Table S1), whereas theanine showed no directional consistency (4/8 higher; 4/8 lower; exact sign test *P* = 1.0). Within catechin subclasses, EC and catechin gallate (CG) were lower in Menghai in 8/8 pairs (Table S1). In parallel, electronic-tongue readouts (available for 7/8 pairs) indicated that bitterness and astringency were lower in Menghai in 7/7 pairs (exact sign test *P* = 0.0156; Table S1), aligning sensory directionality with the subset of strictly shifting chemical components.

Taken together, these results indicate a two-tier pattern under genotype matching. A comparatively stable phenolic/alkaloid component (e.g., EGCG, caffeine) shifts consistently across pairs, whereas theanine varies in direction across genotypes. This framing is interpretive rather than causal: it posits that distinct biochemical subsystems may differ in how tightly their allocation is canalized across genotypes under the same regional contrast. In this context, the Growth–Differentiation Balance Hypothesis offers a useful conceptual language for why carbon-based secondary metabolism can shift systematically under environmental constraints, while acknowledging that the present dataset does not identify the proximal drivers (Herms & Mattson, 1992).

For tea specifically, theanine provides a plausible source of genotype-dependent variation because it functions as a mobile nitrogen pool and is influenced by nitrogen turnover as well as plant-side uptake, assimilation, and transport. Mechanistic work indicates that root microbiota can influence nitrogen homeostasis and theanine synthesis in tea plants (Xin et al., 2024), and seasonal transcriptome–metabolome analyses show that theanine-related pathways can be strongly time-structured and context-dependent (Gong et al., 2020). This also helps explain why the sensory pattern can remain directional even when the composite chemical index is not, because perception may track specific subcomponents rather than an omnibus index (Pires et al., 2020; Zou et al., 2018). Because the sign instability of NPI is largely driven by the nitrogenous pool, we next test whether bulk edaphic N stocks provide a pair-level proxy for theanine allocation under the same matched contrasts.

(A) Paired effect magnitude for VSI and NPI expressed as |Cohen's d_z_| (Menghai − Pu’er).

(B) Paired ΔNPI (Menghai − Pu’er) by genotype-pair index shows mixed signs (6/8 negative, 2/8 positive; Wilcoxon signed-rank *P* = 0.1094); dashed line, ΔNPI = 0.

(C) Paired joint shifts in EGCG (mg/g) and theanine (mg/kg) from Pu’er (open) to Menghai (filled), with lines connecting matched pairs.

### 3.3 Limited pair-level mapping from soil N stocks to theanine

Bulk edaphic inventories differed consistently between sites: soil organic matter (OM), hydrolyzable nitrogen (HN), and total nitrogen (TN) were higher in Menghai in all eight genotype-matched pairs (8/8; exact sign test two-sided *P* = 0.0078 for each; Table S1). Soil total potassium (TK) showed the opposite tendency (lower in Menghai in 7/8 pairs; Table S1); the paired Wilcoxon test supported this tendency (two-sided *P* = 0.0156), whereas the exact sign test provided weaker directional evidence (two-sided *P* = 0.0703; Table S1).

Leaf theanine did not track the TN gradient at the pair level. Specifically, ΔTN (Menghai − Pu’er) was positive in all eight pairs, whereas Δtheanine crossed zero (4/8 positive and 4/8 negative), indicating a lack of pairwise directional concordance between bulk TN and theanine (Fig. S1A; Table S1). Consistently, the within-pair association between ΔTN and Δtheanine was weak and non-significant (Spearman ρ = 0.21, *P* = 0.610; Fig. S1A).

Enzyme activities add process-relevant context but similarly resist a single-variable mapping. Within this framework, urease and sucrase activity trends are interpreted as indicators of local transformation potential at the soil–rhizosphere interface, rather than as direct proxies for pair-level theanine allocation or volatile shift magnitude. Urease activity was higher in Pu’er in 7/8 pairs, whereas β-glucosidase was higher in Menghai in 7/8 pairs (Table S1). Sucrase showed no monotonic association with the magnitude of the volatile shift (ΔSucrase vs ΔVSI: Spearman ρ = −0.05, *P* = 0.911; Fig. S1B). At this resolution, neither bulk stocks nor individual enzyme assays provide a direct pairwise bridge from edaphic enrichment to leaf chemical endpoints.

These results indicate that bulk TN stock is not a sufficient pair-level proxy for theanine allocation in the present spring-flush snapshot. A more plausible interpretation is that leaf nitrogenous outcomes depend on plant-available pools and turnover at the rhizosphere interface rather than on bulk stock alone.

This interpretation is consistent with current views of tea rhizosphere function, in which microbially mediated nutrient transformation at the root–soil interface may be more relevant to plant outcomes than bulk soil inventories alone (Chen et al., 2023). It is also compatible with evidence that tea root microbiota can influence nitrogen homeostasis and theanine synthesis (Xin et al., 2024).

The enzyme results provide process-relevant context, but at this resolution they do not support a simple one-enzyme–one-trait mapping. We therefore turn to rhizosphere community composition to test whether the microbiome itself provides a more pair-robust regional fingerprint beyond what is captured by bulk edaphic proxies.

### 3.4 A robust regional microbiome fingerprint under genotype matching

**Fig. 3:**
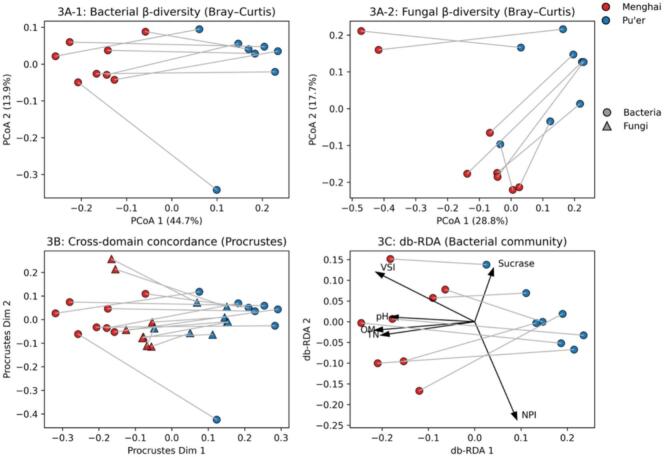
Rhizosphere community structuring under genotype-matched pairing (*n* = 8 pairs).

Given the limited pair-level mapping from bulk edaphic stocks and single-enzyme readouts to nitrogenous leaf endpoints, we next asked whether rhizosphere community composition itself provides a pair-robust regional fingerprint under strict genotype-matched pairing. Both bacterial and fungal communities showed region-associated structuring (Fig. 3A-Fig. 1, Fig. 3A-2). Based on Bray-Curtis dissimilarities, PERMANOVA attributed pseudo-R^2^ = 0.4022 of variance to region in bacteria and 0.2065 in fungi, with significance assessed using an exact pair-restricted permutation scheme (2^8^ = 256 within-pair label swaps; p_pair_ = 0.0117 for both; Table S3). For completeness, the corresponding unrestricted 999-permutation *P*-values are reported as a descriptive reference (P_999_ = 0.001; Table S3), whereas p_pair_ is used as the primary inferential statistic to remain aligned with the paired design.

To ensure that the PERMANOVA signal reflects a shift in centroid location rather than differences in multivariate spread, we evaluated dispersion under the same pair-restricted scheme. Dispersion tests did not provide evidence for region-associated differences in spread (betadisper p_pair_ = 0.2451 for bacteria; 0.3463 for fungi; Table S3), supporting the interpretation that the observed separation is attributable to a location shift in community composition.

At the within-pair level, α-diversity showed directional tendencies that were consistent but not cohort-significant at conventional thresholds. Bacterial Shannon was lower in Menghai in 7/8 pairs (directionality *P* = 0.0703), and fungal Shannon and observed OTUs were each lower in Menghai in 7/8 pairs (directionality P = 0.0703 for both; Table S3). These results are therefore reported as directional tendencies rather than cohort-level shifts.

Beyond single-domain separation, ordination geometry was concordant across kingdoms. Procrustes analysis indicated significant cross-domain agreement between bacterial and fungal ordinations (m^2^ = 0.4064, p_pair_ = 0.0039; Fig. 3B; Table S3; P_999_ = 0.001 shown for reference). For bacteria, db-RDA further indicated that the measured covariate set aligns with the observed compositional structure (adjusted R^2^ = 0.3617, p_pair_ = 0.0039; Fig. 3C; Table S3; standardized variables). The displayed covariates include edaphic properties (e.g., pH, OM, TN), soil enzyme activity (sucrase), and chemistry indices (VSI, NPI); this result is presented as multivariate alignment rather than attribution to any single driver.

Two features strengthen this evidence beyond a generic statement of regional difference. First, inference is explicitly aligned with the genotype-matched design via exact within-pair label swaps, reducing the likelihood that the observed separation is explained by cultivar identity alone. Second, the cross-domain concordance indicates that bacterial and fungal communities shift in a coordinated geometric manner under the same regional contrast, consistent with shared environmental filtering and structured site context.

These findings are compatible with the microbial terroir framework developed in perennial crop systems, where microbiome structure covaries with regional characteristics and can align with metabolome-level differences (Bokulich et al., 2016; Gilbert et al., 2014). Consistent with our definition of microbial terroir as region-structured rhizosphere fingerprints observed under standardized spring-flush harvesting and processing, we treat these results as robust association-level fingerprints rather than causal claims. In tea, current syntheses similarly emphasize that rhizosphere microbiota are shaped jointly by edaphic context and host filtering, and that mechanistic resolution requires moving from compositional association to functional testing (Chen et al., 2023).

To connect the community-level separation to interpretable taxa without overfitting, representative genus-level summaries are provided as descriptive context (Fig. S2A). The paired juxtaposition of ΔVSI with ΔPaenibacillus (Fig. S2B) illustrates that pairwise effect magnitudes can vary across genotypes.

At the genus level, the present data suggest a layered pattern rather than a single one-to-one microbial explanation: some candidate taxa show broadly directional regional tendencies across genotype-matched pairs, whereas others appear more genotype-contingent in their pairwise behavior. In the present dataset, the most robust terroir-related microbiome features lie at the level of community structure and cross-kingdom concordance, whereas genus-level patterns are better interpreted as candidate regional correlates than as individually sufficient terroir markers. Therefore, we next test whether the multi-layer signal remains recoverable when generalization is forced across genotype-matched pairs using LOPO validation with pair-respecting inference (Section 3.5).

(A) Bray–Curtis PCoA for bacterial (A1) and fungal (A2) communities; grey lines connect matched pairs (PERMANOVA with pair-restricted permutations, 2^8^ swaps: bacteria pseudo-R^2^ = 0.4022, p_pair_ = 0.0117; fungi pseudo-R^2^ = 0.2065, p_pair_ = 0.0117).

(B) Procrustes concordance between bacterial and fungal ordinations (m^2^ = 0.4064; p_pair_ = 0.0039).

(C) db-RDA of the bacterial community with edaphic and chemical covariates (adjusted R^2^ = 0.3617; p_pair_ = 0.0039).

### 3.5 Cross-pair recoverability under LOPO with an exact pair-respecting null

**Fig. 4:**
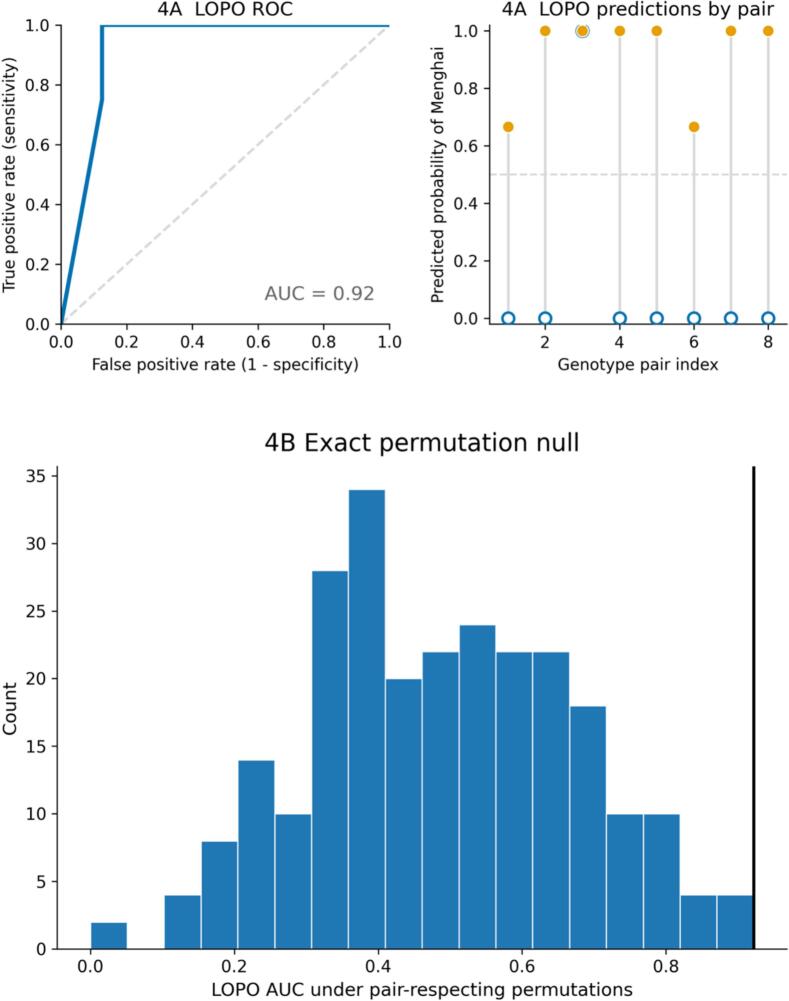
Cross-pair recoverability of the regional signature under LOPO evaluation (*n* = 8 pairs).

To assess whether the regional contrast remains recoverable when generalization is forced across genotype-matched pairs, we performed leave-one-pair-out (LOPO) cross-validation using a random-forest classifier (Breiman, 2001). The integrated feature panel produced an out-of-fold ROC AUC of 0.92 across eight LOPO folds (Fig. 4A). Pair-level predictions indicated that most held-out pairs were separated with high confidence, while a small number of pairs exhibited intermediate probabilities (Fig. 4A, right), consistent with an overall AUC below 1.0.

To evaluate whether this performance could arise by chance under the matched design, we conducted an exact pair-respecting permutation test by enumerating all 2^8^ = 256 within-pair label swaps and recomputing LOPO AUC for each relabeling (Fig. 4B). The observed AUC lay in the upper tail of the exact null distribution, yielding an exact permutation *p*-value of 0.0195 with the standard +1 adjustment (Phipson & Smyth, 2010).

LOPO directly targets a key vulnerability of small-n paired omics, where optimistic discrimination can occur if models implicitly exploit pair-specific structure. By treating the matched pair as the unit of generalization, LOPO requires that separation be supported by signals that recur across pairs rather than pair-specific variation tied to particular genotypes. The exact, pair-respecting permutation further aligns inference with the dependence structure by preserving within-pair feature distributions while removing systematic region–feature alignment (Edgington & Onghena, 2007; Ojala & Garriga, 2010). Given the discretized nature of ROC/AUC under two-sample test folds, these results are interpreted as evidence of cross-pair recoverability under the present panel and pipeline, rather than as a stable estimate of population-level predictive performance (Fawcett, 2006; Varma & Simon, 2006).

(A) LOPO ROC for the integrated feature panel (AUC = 0.92), with paired out-of-fold predicted probabilities shown by genotype pair (right).

(B) Exact pair-respecting permutation null for LOPO AUC generated by enumerating all 2^8^ within-pair label swaps (256 permutations); the vertical line marks the observed AUC (exact permutation *P* = 0.0195).

### 3.6 Mechanistic interpretation and study boundaries

Across layers, the clearest pair-consistent signal under strict genotype matching lies in the volatile domain. VSI is higher in Menghai in every pair (8/8), and β-caryophyllene shows a pipeline-defined detect/non-detect asymmetry (detected in Pu’er in 7/8 pairs; below the detection rule in all matched Menghai samples, 0/8).

The non-volatile layer was more partitioned: EGCG and caffeine were consistently higher in Menghai (8/8 each), whereas theanine varied in direction across genotypes (4/8 higher; 4/8 lower), accounting for the lack of pair-invariant behavior of NPI. Bulk edaphic stocks show a parallel limitation: TN was higher in Menghai in all pairs (8/8), yet leaf theanine does not rise in a matched manner, and ΔTN does not track Δtheanine (Spearman ρ = 0.21, *P* = 0.610). In parallel, both bacterial and fungal communities exhibit regional β-diversity separation with cross-kingdom concordance, and the integrated multi-layer signal remained recoverable under LOPO evaluation.

These results support a bounded interpretation rather than a causal one. The co-occurrence of a uniform VSI shift and a β-caryophyllene detect/non-detect contrast is compatible with region-associated differences in sesquiterpene-related chemistry and/or defence-associated processes, but it is also compatible with a purely analytical explanation in which β-caryophyllene in Menghai falls below the current detection/blank-filtering rule. Accordingly, the contrast is best regarded as an operational marker defined by the present pipeline rather than as direct evidence of biochemical on/off regulation.

The TN–theanine decoupling likewise limits nitrogen-stock narratives. The paired data do not support bulk soil TN stock as a sufficient determinant of leaf theanine allocation in this spring-flush snapshot. A more adequate explanation must include intermediate layers that vary independently of bulk stocks, including plant-available N pools and turnover at the rhizosphere interface, rhizosphere microsite processes averaged out in bulk assays, and plant-side control of uptake, assimilation, transport, and remobilization.

Boundary conditions should therefore remain explicit. The data reflect a single season and phenological window (spring flush) under standardized green-tea processing. The β-caryophyllene contrast is defined relative to a fixed detection/blank-filtering pipeline, and near-threshold detectability may shift with sensitivity and preprocessing. Bulk edaphic metrics (TN, OM, HN, TK) are stock measures and may not capture the N forms and short-term dynamics most relevant to leaf allocation, while single enzyme assays provide limited coverage of rate-limiting steps. Taxon-based functional prediction methods (e.g., PICRUSt2 and FUNGuild) provide inferred functional potential from taxonomic composition, but they do not directly measure in situ gene expression, enzyme activity, or realized process rates. These predictions are interpreted here as exploratory contextual support rather than as direct mechanistic evidence for microbiome-mediated flavour differences. With *n* = 8 pairs, the design supports statements about paired directionality and cross-pair recoverability, but it does not support definitive causal decomposition.

This framing motivates directly testable follow-up work. Future studies should evaluate the transportability of the paired VSI shift and the β-caryophyllene contrast across independent harvests and years, test whether the current β-caryophyllene contrast collapses into a continuous low-abundance distribution under more sensitive targeted quantification, and replace bulk stock measures with flux-related indices more directly linked to nitrogen allocation.

## 4 Conclusions

By employing a strict genotype-matched design (n = 8 pairs) during a controlled spring-flush window, we identified a recoverable multi-layer regional contrast across matched genotypes.

Volatile profiling revealed the clearest regional signal. The Volatile Signature Index (VSI) was consistently higher in Menghai across all matched pairs. In addition β-caryophyllene showed a distinct detect/non-detect contrast under the current analytical pipeline. In the non-volatile layer, EGCG and caffeine shifted consistently, whereas theanine varied by genotype, such that the composite NPI did not show a pair-invariant direction.

Bulk edaphic stocks were limited pair-level proxies for leaf nitrogenous outcomes. Although OM, HN, and TN were uniformly higher in Menghai, bulk TN did not track leaf theanine. By contrast, rhizosphere bacterial and fungal communities showed clear region-associated differentiation under the matched design, and the integrated chemical and microbial signal remained recoverable under LOPO evaluation.

These findings support an association-level interpretation of spring-flush regional flavour signatures and their rhizosphere microbial correlates under genotype control. The present study does not establish a definitive causal microbiome–flavour relationship, and its inferences remain bounded by a single seasonal window and the matched composite-pair sampling framework. Future work should prioritize multi-season validation, targeted quantification of near-threshold volatile markers, and more direct measurements of nitrogen-related flux and plant-side regulation.

## CRediT authorship contribution statement

**Jiayin Tong:** Resources. **Yunhan Li:** Formal analysis, Data curation. **Yanmei Zhang:** Resources. **Panpan Zhang:** Funding acquisition. **Kaibo Wang:** Funding acquisition. **Qian Zou:** Writing – review & editing, Writing – original draft. **Shiquan Shen:** Writing – review & editing, Conceptualization.

## Funding

This work was supported by the Basic Research Special Project of the Yunnan Provincial Department of Science and Technology [202501AT070080]; Central funds guiding local scientific and technological development [202407AB110014].

## Declaration of competing interest

The authors declare that they have no known competing financial interests or personal relationships that could have appeared to influence the work reported in this paper.

## Data Availability

Data will be made available on request.

## Appendix A Supplementary data

Supplementary data associated with this article include Supplementary Tables S1–S3 and Supplementary Figures S1–S2 (panels A–B).
