# Supplementary material for: Genotype-matched mapping reveals consistent regional flavour signatures and rhizosphere microbial correlates in spring-flush Yunnan large-leaf tea
Source: Food Chem X. 2026 Apr 16;35:103869. doi: 10.1016/j.fochx.2026.103869 (PMC13103584; doi:10.1016/j.fochx.2026.103869)
Supplement: Supplementary file 1 — Supplementary data associated with this article include Supplementary Tables S1–S3 and Supplementary Figures S1–S2 (panels A–B). [file mmc1.docx]

Supporting Information for Publication

Jiayin Tong^a,b,c,1^, Yunhan Li^d,1^, Yanmei Zhang^a,b,c^, Panpan Zhang^a,b,c^,Kaibo Wang^a,b,c^,Qian Zou^a*^,Shiquan Shen^a,b,c*^


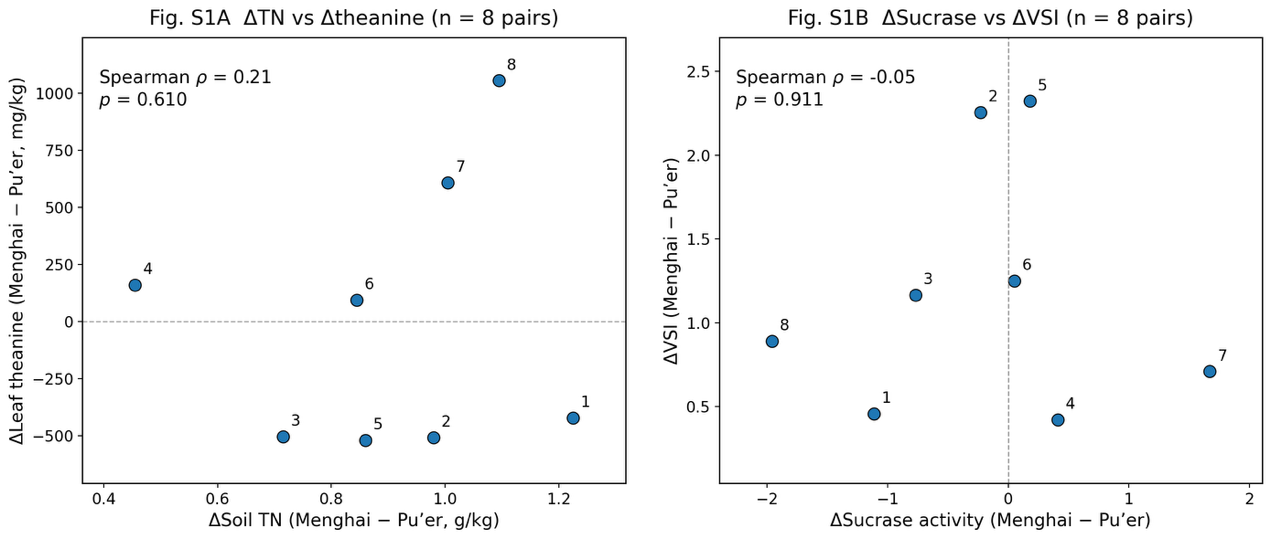


Figure S1. Pairwise associations between bulk edaphic variables and leaf chemical endpoints under strict genotype matching (n = 8 pairs).

(A) Relationship between within-pair differences in bulk soil total nitrogen (ΔTN; Menghai − Pu’er) and leaf theanine (Δtheanine; Menghai − Pu’er).

(B) Relationship between within-pair differences in soil sucrase activity (ΔS-SC; Menghai - Pu’er) and the Volatile Signature Index (ΔVSI; Menghai - Pu’er).

Points are labeled by genotype pair index (1-8). Spearman’s rank correlation (ρ) and two-sided p-values are shown. Dashed lines indicate zero difference.


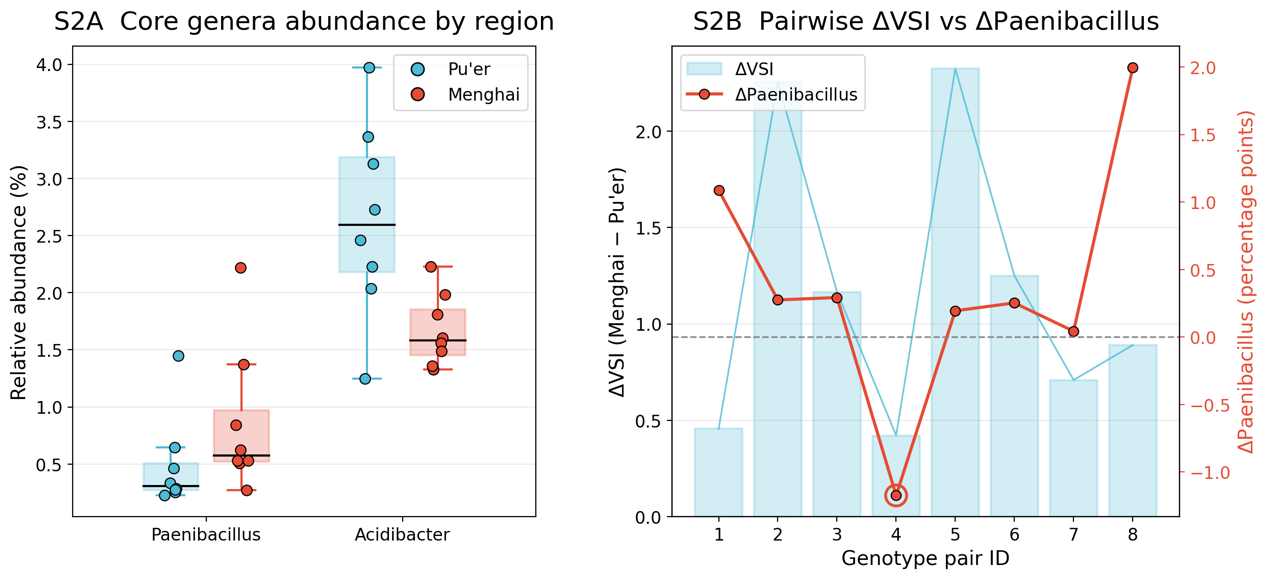


Figure S2. Taxon-level summaries and an illustrative pairwise juxtaposition (n = 8 pairs).

(A) Relative abundance (%) of selected genera by region (Pu’er vs Menghai), shown as boxplots with individual samples overlaid.

(B) Paired ΔVSI (bars; Menghai − Pu’er) plotted alongside paired ΔPaenibacillus (line; Menghai − Pu’er) by genotype-pair ID; dashed line indicates ΔVSI = 0.

**Table S1. Summary statistics for genotype-matched pairwise differences (Menghai − Pu’er; n = 8 pairs).**

| Target Metric | Layer | Data Type | Directional Consistency | n (pairs used) | Median Δ (M−P) | IQR Δ | Range Δ (min, max) | Paired dz | Bootstrap 95% CI (dz) | Exact sign test p | Wilcoxon p | Pairedt-test p | McNemar exact p | McNemar discordant (n01,n10) | Notes |
| --- | --- | --- | --- | --- | --- | --- | --- | --- | --- | --- | --- | --- | --- | --- | --- |
| Volatile layer (index & key compounds) | | | | | | | | | | | | | | | |
| VSI | Volatile (Index) | Continuous | 8 >, 0 <, 0 = across 8 pairs | 8 | 1.028 | [0.646, 1.5] | [0.422, 2.32] | 1.592 | [1.25, 3.02] | 0.0078125 | 0.0078125 | 0.0027859 |  |  | Δ = VSI(Menghai) − VSI(Pu’er). dz and bootstrap CI reported in Fig.1B. |
| Linalool (Area%) | Volatile (Compound) | Continuous | 8 >, 0 <, 0 = across 8 pairs | 8 | 3.643 | [1.37, 6.67] | [0.09, 16.9] | 0.941 |  | 0.0078125 | 0.0078125 | 0.0323705 |  |  | CAS 78-70-6; Area% averaged across technical replicates; non-detects treated as 0 (none observed). |
| β-Caryophyllene presence | Volatile (Compound) | Binary (detect/non-detect) | 7/8 pairs: detected in Pu’er only (M absent, P detected); 1/8 both absent | 8 |  |  |  |  |  |  |  |  | 0.0156250 | (7,0) | CAS 87-44-5; detect defined as Area% > 0 after pipeline; McNemar exact uses discordant pairs (n01=7, n10=0). |
| β-Caryophyllene (Area%, zero-filled) | Volatile (Compound) | Continuous (zero-filled) | 0 >, 7 <, 1 = across 8 pairs | 8 | -0.090 | [-0.105, -0.075] | [-0.13, 0] | -2.076 |  | 0.0156250 | 0.0177559 | 0.0006160 |  |  | CAS 87-44-5; Area% across technical replicates in Pu’er when detected: 0.05–0.13; sample mean range: [0.06, 0.13]. |
| 1-Octen-3-ol (Area%, zero-filled) | Volatile (Compound) | Continuous (zero-filled) | 1 >, 6 <, 1 = across 8 pairs | 8 | -0.190 | [-0.351, -0.124] | [-0.71, 0.48] | -0.564 |  | 0.1250000 | 0.1281902 | 0.1548152 |  |  | CAS 3391-86-4; non-detects treated as 0 to match preprocessing used for indices. |
| Non-volatile leaf chemistry | | | | | | | | | | | | | | | |
| NPI | Non-volatile (Index) | Continuous | 2 >, 6 <, 0= across 8 pairs | 8 | -1.160 | [-2.04, -0.564] | [-2.23, 1.57] | -0.685 |  | 0.2890625 | 0.1093750 | 0.0937062 |  |  | Δ = NPI(Menghai) − NPI(Pu’er). |
| EGCG (mg/g) | Non-volatile (Catechin) | Continuous | 8 >, 0 <, 0= across 8 pairs | 8 | 1.576 | [1.43, 1.93] | [1.17, 2.25] | 4.653 |  | 0.0078125 | 0.0078125 | 0.0000034 |  |  |  |
| caffeine (mg/g) | Non-volatile (Alkaloid) | Continuous | 8 >, 0 <, 0= across 8 pairs | 8 | 0.420 | [0.199, 0.671] | [0.137, 0.965] | 1.450 |  | 0.0078125 | 0.0078125 | 0.0045712 |  |  |  |
| theanine (mg/kg) | Non-volatile (Amino acid) | Continuous | 4 >, 4 <, 0= across 8 pairs | 8 | -163.363 | [-504, 272] | [-519, 1.05e+03] | -0.007 |  | 1.0000000 | 1.0000000 | 0.9850149 |  |  |  |
| EC (mg/g) | Non-volatile (Catechin) | Continuous | 0 >, 8 <, 0= across 8 pairs | 8 | -0.346 | [-0.418, -0.248] | [-0.505, -0.102] | -2.306 |  | 0.0078125 | 0.0078125 | 0.0003274 |  |  |  |
| CG (mg/g) | Non-volatile (Catechin) | Continuous | 0 >, 8 <, 0= across 8 pairs | 8 | -0.197 | [-0.236, -0.123] | [-0.438, -0.0063] | -1.554 |  | 0.0078125 | 0.0078125 | 0.0031722 |  |  | CG = catechin gallate (mg/g). |
| Soil stocks | | | | | | | | | | | | | | | |
| Soil organic matter, OM | Soil (Stock) | Continuous | 8 >, 0 <, 0= across 8 pairs | 8 | 20.075 | [16.6, 23.3] | [8.45, 31.6] | 2.869 |  | 0.0078125 | 0.0078125 | 0.0000833 |  |  |  |
| Soil hydrolyzable N, HN | Soil (Stock) | Continuous | 8 >, 0 <, 0= across 8 pairs | 8 | 98.250 | [82.2, 118] | [64, 133] | 3.987 |  | 0.0078125 | 0.0078125 | 0.0000096 |  |  |  |
| Soil total N, TN | Soil (Stock) | Continuous | 8 >, 0 <, 0= across 8 pairs | 8 | 0.920 | [0.812, 1.03] | [0.455, 1.22] | 3.764 |  | 0.0078125 | 0.0078125 | 0.0000141 |  |  |  |
| Soil total K, TK | Soil (Stock) | Continuous | 1 >, 7 <, 0= across 8 pairs | 8 | -6.778 | [-8.06, -3.71] | [-8.78, 0.57] | -1.589 |  | 0.0703125 | 0.0156250 | 0.0028136 |  |  |  |
| Soil enzymes | | | | | | | | | | | | | | | |
| Urease activity (S-UE) | Soil (Enzyme) | Continuous | 1 >, 7 <, 0= across 8 pairs | 8 | -87.600 | [-205, -9.38] | [-345, 64.6] | -0.785 |  | 0.0703125 | 0.0546875 | 0.0618650 |  |  |  |
| β-Glucosidase activity (S-β-GC) | Soil (Enzyme) | Continuous | 7 >, 1 <, 0= across 8 pairs | 8 | 298.500 | [126, 370] | [-558, 406] | 0.560 |  | 0.0703125 | 0.1953125 | 0.1569585 |  |  |  |
| Sucrase activity (S-SC) | Soil (Enzyme) | Continuous | 4 >, 4 <, 0= across 8 pairs | 8 | -0.090 | [-0.856, 0.237] | [-1.96, 1.67] | -0.202 |  | 1.0000000 | 0.6406250 | 0.5852573 |  |  |  |
| Sensory (E-tongue) | | | | | | | | | | | | | | | |
| E-tongue sourness | Sensory (E-tongue) | Continuous | 0 >, 7 <, 0= across 7 pairs | 7 | -8.640 | [-9.69, -7.75] | [-12.8, -1.57] | -2.410 |  | 0.0156250 | 0.0156250 | 0.0006991 |  |  | Pair 5 excluded due to missing Menghai sensory values. |
| E-tongue bitterness | Sensory (E-tongue) | Continuous | 0 >, 7 <, 0= across 7 pairs | 7 | -1.550 | [-3.1, -0.99] | [-5.15, -0.65] | -1.348 |  | 0.0156250 | 0.0156250 | 0.0118232 |  |  | Pair 5 excluded due to missing Menghai sensory values. |
| E-tongue astringency | Sensory (E-tongue) | Continuous | 0 >, 7 <, 0= across 7 pairs | 7 | -3.450 | [-6.13, -2.97] | [-8.79, -1.3] | -1.754 |  | 0.0156250 | 0.0156250 | 0.0035408 |  |  | Pair 5 excluded due to missing Menghai sensory values. |
| E-tongue saltiness | Sensory (E-tongue) | Continuous | 7 >, 0 <, 0= across 7 pairs | 7 | 9.980 | [7.76, 11.1] | [3.54, 13.7] | 2.818 |  | 0.0156250 | 0.0156250 | 0.0003003 |  |  | Pair 5 excluded due to missing Menghai sensory values. |
| E-tongue richness | Sensory (E-tongue) | Continuous | 0 >, 7 <, 0= across 7 pairs | 7 | -4.470 | [-6.37, -3.88] | [-8.31, -2.03] | -2.301 |  | 0.0156250 | 0.0156250 | 0.0008943 |  |  | Pair 5 excluded due to missing Menghai sensory values. |
|  |  |  |  |  |  |  |  |  |  |  |  |  |  |  |  |
| *Notes: Δ denotes within-pair difference (Menghai − Pu’er). Exact sign test is computed on non-zero Δ values (zeros treated as ties). For volatile Area% features, compound-level values are averaged across technical replicates; non-detects are treated as 0 where indicated to match the preprocessing used for indices and paired dz calculations.* | | | | | | | | | | | | | | | |

| Table S2. Top 20 cleaned volatile features ranked by \|paired Cohen's dz\| (Menghai − Pu’er). | | | | | | | | | | | |
| --- | --- | --- | --- | --- | --- | --- | --- | --- | --- | --- | --- |
|  |  |  |  |  |  |  |  |  |  |  |  |
| CAS No. | **Compound (tentative identification)** | **Abbrev.** | **MH mean ± SD (Area%)** | **PR mean ± SD (Area%)** | **Mean Δ (MH−PR) (Area%)** | **Paired dz** | **n(Δ>0)** | **n(Δ<0)** | **n(Δ=0)** | **Detected in PR (n/8)** |  |
| 14901-07-6 | 3-Buten-2-one, 4-(2,6,6-trimethyl-1-cyclohexen-1-yl)- | β-Ionone | 0.227 ± 0.063 | 0.026 ± 0.049 | 0.20125 | 2.4616142 | 8 | 0 | 0 | 2 |  |
| 110-42-9 | Methyl decanoate (Decanoic acid, methyl ester) | MeDec | 0.838 ± 0.345 | 0.283 ± 0.177 | 0.555 | 2.2957641 | 8 | 0 | 0 | 8 |  |
| 87-44-5 | Caryophyllene | β-Caryo | 0.000 ± 0.000 | 0.084 ± 0.040 | -0.08375 | -2.076441 | 0 | 7 | 1 | 7 |  |
| 464-17-5 | Bicyclo[2.2.1]hept-2-ene, 1,7,7-trimethyl- | TM-Bicyclo[2.2.1] | 0.014 ± 0.039 | 0.271 ± 0.152 | -0.2575 | -1.941373 | 0 | 7 | 1 | 7 |  |
| 106-70-7 | Hexanoic acid, methyl ester | MeHex | 0.831 ± 0.537 | 0.062 ± 0.177 | 0.76875 | 1.4001138 | 6 | 0 | 2 | 1 |  |
| 496-16-2 | Benzofuran, 2,3-dihydro- | 2,3-DHBF | 0.000 ± 0.000 | 0.129 ± 0.096 | -0.12875 | -1.347158 | 0 | 6 | 2 | 6 |  |
| 111-11-5 | Octanoic acid, methyl ester | MeOct | 0.649 ± 0.468 | 0.078 ± 0.219 | 0.57125 | 1.1263833 | 8 | 0 | 0 | 1 |  |
| 78-70-6 | Linalool | Linalool | 14.246 ± 5.552 | 8.859 ± 2.089 | 5.3875 | 0.9411784 | 8 | 0 | 0 | 8 |  |
| 112-31-2 | Decanal | Decanal | 0.774 ± 0.211 | 0.497 ± 0.209 | 0.276875 | 0.9181944 | 6 | 2 | 0 | 8 |  |
| 488-10-8 | 2-Cyclopenten-1-one, 3-methyl-2-(2-pentenyl)-, (Z)- | Z-MeCPenone | 0.368 ± 0.276 | 0.123 ± 0.111 | 0.244375 | 0.8912101 | 5 | 3 | 0 | 7 |  |
| 3338-55-4 | 1,3,6-Octatriene, 3,7-dimethyl-, (Z)- | Z-DMOT | 2.218 ± 1.134 | 3.699 ± 1.530 | -1.480625 | -0.882926 | 1 | 7 | 0 | 8 |  |
| 111-82-0 | Methyl dodecanoate (Dodecanoic acid, methyl ester) | MeDod | 0.449 ± 0.109 | 0.283 ± 0.195 | 0.16625 | 0.805771 | 6 | 2 | 0 | 8 |  |
| 29957-43-5 | 1,5,7-Octatrien-3-ol, 3,7-dimethyl- | Hotrienol | 4.571 ± 1.937 | 8.336 ± 5.562 | -3.764375 | -0.786476 | 1 | 7 | 0 | 8 |  |
| 432-25-7 | 1-Cyclohexene-1-carboxaldehyde, 2,6,6-trimethyl- | β-Cyclocitral | 0.137 ± 0.118 | 0.048 ± 0.089 | 0.089375 | 0.782488 | 4 | 1 | 3 | 2 |  |
| 119-36-8 | Methyl salicylate | MeSA | 0.397 ± 0.398 | 0.855 ± 0.822 | -0.4575 | -0.748751 | 3 | 5 | 0 | 6 |  |
| 123-35-3 | .beta.-Myrcene | β-Myrcene | 2.732 ± 1.521 | 4.632 ± 2.888 | -1.899375 | -0.732827 | 1 | 7 | 0 | 8 |  |
| 17092-92-1 | 2(4H)-Benzofuranone, 5,6,7,7a-tetrahydro-4,4,7a-trimethyl-, (R)- | 2(4H)-Benzofuranon… | 0.128 ± 0.184 | 0.000 ± 0.000 | 0.128125 | 0.6952983 | 3 | 0 | 5 | 0 |  |
| 52957-16-1 | 9-Tetradecen-1-ol, (E)- | 9-Tetradecen-1-ol,… | 0.142 ± 0.205 | 0.000 ± 0.000 | 0.1425 | 0.6951515 | 3 | 0 | 5 | 0 |  |
| 3796-70-1 | 5,9-Undecadien-2-one, 6,10-dimethyl-, (E)- | 5,9-Undecadien-2-o… | 0.389 ± 0.076 | 0.315 ± 0.078 | 0.074375 | 0.6945353 | 6 | 2 | 0 | 8 |  |
| 562-74-3 | Terpinen-4-ol | Terpinen-4-ol | 0.167 ± 0.260 | 0.000 ± 0.000 | 0.1675 | 0.6439927 | 3 | 0 | 5 | 0 |  |

**Table S3. Genotype-matched microbiome results (n = 8 pairs).**

| Analysis | Domain | Distance / Ordination | Test statistic | Statistic value | df1 | df2 | p (unrestricted, N=999) | p_pair (within-pair, 2^8 exact) | Permutations (unrestricted) | Permutations(pair-respecting) |  |  |
| --- | --- | --- | --- | --- | --- | --- | --- | --- | --- | --- | --- | --- |
| PERMANOVA (region effect) | Bacteria | Bray–Curtis | pseudo-F | 9.4211 | 1 | 14 | 0.0010 | 0.0117 | 999 | 2^8^ exact (within-pair) | | |
| PERMANOVA (region effect) | Fungi | Bray–Curtis | pseudo-F | 3.6433 | 1 | 14 | 0.0010 | 0.0117 | 999 | 2^8^ exact (within-pair) | | |
| PERMANOVA (region effect) | Bacteria | Bray–Curtis | pseudo-R² | 0.4022 |  |  |  |  | 999 | 2^8^ exact (within-pair) | | |
| PERMANOVA (region effect) | Fungi | Bray–Curtis | pseudo-R² | 0.2065 |  |  |  |  | 999 | 2^8^ exact (within-pair) | | |
| betadisper (dispersion) | Bacteria | Bray–Curtis (PCoA) | F | 1.4306 | 1 | 14 | 0.2700 | 0.2451 | 999 | 2^8^ exact (within-pair) | | |
| betadisper (dispersion) | Fungi | Bray–Curtis (PCoA) | F | 1.1566 | 1 | 14 | 0.3060 | 0.3463 | 999 | 2^8^ exact (within-pair) | | |
| Alpha diversity (directionality) | Bacteria | Shannon index | Exact sign test (two-sided) | 7/8 lower in Menghai (Δ<0) | | | | 0.0703 |  | 2^8^ exact (within-pair) | | |
| Alpha diversity (directionality) | Fungi | Shannon index | Exact sign test (two-sided) | 7/8 lower in Menghai (Δ<0) | | | | 0.0703 |  | 2^8^ exact (within-pair) | | |
| Alpha diversity (directionality) | Fungi | Observed OTUs | Exact sign test (two-sided) | 7/8 lower in Menghai (Δ<0) | | | | 0.0703 |  | 2^8^ exact (within-pair) | | |
| Procrustes (cross-domain concordance) | Bacteria vs Fungi | PCoA axes 1–2 | m^2^ (disparity) | 0.4064 |  |  | 2^8^ exact (within-pair) | 0.0039 | 999 | 2^8^ exact (within-pair) | | |
| db-RDA (global model) | Bacteria | Bray–Curtis → PCoA (all + eigen) → multivariate regression | pseudo-F | 2.4164 | 6 | 9 | 0.0010 | 0.0039 | 999 | 2^8^ exact (within-pair) | | |
| db-RDA (global model) | Bacteria | Covariates: pH, OM, TN, Sucrase, VSI, NPI(z-scored) | R²_adj_ | 0.3617 |  |  |  |  | 999 | 2^8^ exact (within-pair) | | |
| db-RDA (global model) | Bacteria | Covariates: pH, OM, TN, Sucrase, VSI, NPI(z-scored) | R² | 0.6170 |  |  |  |  | 999 | 2^8^ exact (within-pair) | | |

Table S4. Sample-level quantitative data for non-volatile metabolites for each cultivar × site.

| Sample | NPI (unitless) | theanine (mg/kg) | GABA (mg/kg) | caffeine (mg/g) | GA (mg/g) | GC (mg/g) | EGC (mg/g) | C (mg/g) | EC (mg/g) | EGCG (mg/g) | GCG (mg/g) | ECG (mg/g) | CG (mg/g) |
| --- | --- | --- | --- | --- | --- | --- | --- | --- | --- | --- | --- | --- | --- |
| M1 | 1.835 | 2016.025 | 6.225 | 3.745 | 0.053 | 0.571 | 2.823 | 0.408 | 0.864 | 8.974 | 1.160 | 2.812 | 0.115 |
| P1 | 2.897 | 2437.490 | 6.290 | 3.537 | 0.105 | 0.490 | 1.986 | 0.533 | 0.967 | 7.806 | 1.213 | 3.751 | 0.344 |
| M2 | -0.447 | 1633.175 | 1.435 | 3.309 | 0.019 | 0.765 | 3.218 | 0.689 | 1.689 | 6.847 | 0.291 | 3.685 | 0.178 |
| P2 | 1.786 | 2139.880 | 2.780 | 3.034 | 0.035 | 0.773 | 3.373 | 0.754 | 1.966 | 5.458 | 0.177 | 3.819 | 0.293 |
| M3 | -3.033 | 765.085 | 1.375 | 3.678 | 0.050 | 0.492 | 2.651 | 0.950 | 2.737 | 5.378 | 0.762 | 4.385 | 0.224 |
| P3 | -0.981 | 1268.240 | 1.350 | 3.112 | 0.028 | 0.729 | 2.816 | 0.913 | 3.139 | 3.848 | 0.341 | 4.130 | 0.231 |
| M4 | -4.960 | 1111.580 | 0.885 | 4.606 | 0.094 | 0.787 | 3.152 | 0.867 | 1.876 | 6.173 | 0.842 | 3.902 | 0.139 |
| P4 | -2.931 | 951.525 | 1.155 | 3.711 | 0.153 | 1.242 | 3.358 | 1.142 | 2.247 | 4.190 | 0.373 | 3.524 | 0.578 |
| M5 | 0.184 | 1880.620 | 4.135 | 3.845 | 0.021 | 0.365 | 3.611 | 0.406 | 1.107 | 8.312 | 0.499 | 2.714 | 0.248 |
| P5 | 1.442 | 2399.965 | 3.970 | 3.673 | 0.062 | 0.478 | 2.136 | 0.539 | 1.267 | 6.396 | 0.429 | 3.690 | 0.451 |
| M6 | -0.133 | 1507.900 | 5.390 | 4.152 | 0.029 | 0.223 | 3.167 | 0.480 | 1.096 | 7.916 | 0.831 | 2.993 | 0.135 |
| P6 | 0.815 | 1413.160 | 2.855 | 3.187 | 0.017 | 0.377 | 2.998 | 0.397 | 1.601 | 5.670 | 0.522 | 3.538 | 0.262 |
| M7 | 0.023 | 2278.515 | 3.955 | 4.084 | 0.047 | 0.550 | 4.194 | 0.490 | 1.544 | 6.636 | 0.441 | 2.425 | 0.187 |
| P7 | -0.566 | 1670.210 | 1.765 | 3.488 | 0.076 | 0.867 | 3.273 | 0.694 | 2.011 | 5.194 | 0.432 | 2.928 | 0.445 |
| M8 | 2.818 | 3822.615 | 3.130 | 3.534 | 0.039 | 1.066 | 3.439 | 0.890 | 1.256 | 7.375 | 0.426 | 3.361 | 0.180 |
| P8 | 1.250 | 2767.775 | 2.160 | 3.397 | 0.060 | 1.140 | 3.513 | 0.961 | 1.578 | 5.753 | 0.219 | 3.326 | 0.371 |

**Notes:** Values are reported for each genotype-matched cultivar × site sample (Menghai or Pu’er). Non-volatile analytes include catechins, caffeine, theanine, GABA, and the derived NPI values used in the manuscript. These sample-level data underlie the paired contrasts and summary statistics reported in the main text and supplementary summary tables. Units are shown in the column headers.

Table S5. Sample-level quantitative data for volatile metabolites for each cultivar × site.

| Pair | Cultivar | Site | Sample | VSI (unitless) | linalool (Area%) | gGeraniol (Area%) | decanal (Area%) | linalool oxide (Area%) | β-Caryophyllene (Area%) | 1-Octen-3-ol (Area%) |
| --- | --- | --- | --- | --- | --- | --- | --- | --- | --- | --- |
| 1 | Yunkang-10 | Menghai | M1 | -0.402 | 31.150 | 0.000 | 0.960 | 1.410 | 0.000 | 0.420 |
| 1 | Yunkang-10 | Pu’er | P1 | -0.859 | 25.550 | 2.750 | 0.620 | 9.000 | 0.260 | 1.380 |
| 2 | Xueya-100 | Menghai | M2 | 1.054 | 25.560 | 0.000 | 2.140 | 2.810 | 0.000 | 0.000 |
| 2 | Xueya-100 | Pu’er | P2 | -1.201 | 17.580 | 0.000 | 1.310 | 2.800 | 0.100 | 1.420 |
| 3 | Pujing | Menghai | M3 | 1.098 | 38.460 | 2.230 | 0.520 | 4.940 | 0.000 | 0.280 |
| 3 | Pujing | Pu’er | P3 | -0.068 | 31.350 | 2.370 | 1.520 | 4.430 | 0.180 | 0.980 |
| 4 | Yungui | Menghai | M4 | 1.115 | 29.150 | 1.850 | 1.810 | 2.280 | 0.000 | 0.000 |
| 4 | Yungui | Pu’er | P4 | 0.693 | 25.700 | 2.780 | 0.540 | 9.010 | 0.160 | 0.000 |
| 5 | Zijuan | Menghai | M5 | 0.334 | 21.200 | 2.160 | 0.750 | 2.600 | 0.000 | 0.510 |
| 5 | Zijuan | Pu’er | P5 | -1.988 | 20.840 | 2.610 | 0.500 | 5.260 | 0.240 | 2.210 |
| 6 | Aifeng | Menghai | M6 | 0.813 | 27.760 | 1.290 | 1.010 | 9.160 | 0.000 | 0.880 |
| 6 | Aifeng | Pu’er | P6 | -0.437 | 18.340 | 0.760 | 1.420 | 2.530 | 0.000 | 1.420 |
| 7 | Duanjiebaihao | Menghai | M7 | 0.657 | 51.650 | 1.850 | 0.540 | 3.130 | 0.000 | 0.960 |
| 7 | Duanjiebaihao | Pu’er | P7 | -0.052 | 17.480 | 2.200 | 1.210 | 2.800 | 0.180 | 0.000 |
| 8 | QianMei-601 | Menghai | M8 | 0.067 | 35.570 | 0.000 | 1.190 | 4.260 | 0.000 | 1.180 |
| 8 | QianMei-601 | Pu’er | P8 | -0.823 | 26.280 | 1.200 | 0.830 | 5.550 | 0.120 | 1.510 |

**Notes:** Values are reported for each genotype-matched cultivar × site sample (Menghai or Pu’er). Volatile variables include the compound-level measurements used to construct the Volatile Signature Index (VSI), together with the corresponding sample-level VSI values used in the manuscript. Where applicable, compound-level values represent final processed values after averaging across technical replicates under the same preprocessing pipeline used for the paired analyses in the manuscript. Units or relative abundance metrics are shown in the column headers.
